# 

**Published:** 1872-04

**Authors:** 


### CONTENTS:

### ORIGINAL COMMUNICATIONS.

ARTICLE I.—Class Address. By T. H. Fergnson, D.D.S. .... 529

ARTICLE II.—The Use (not abuse) of Nitrous Oxide Gas. By Arthur C. Ford, D.D.S. .... 531

ARTICLE III.—Reply to Rubber Company's Chemical Advocate. By J. R. Walker, D.D.S. 537

ARTICLE IV.—Eastern Ohio Dental Association. By Dr. D. B. McLain .... 540

ARTICLE V.—Extracts from an Address on Dental Surgery. Delivered by Oliver W. Holmes, M. D. .... 542

### SELECTED ARTICLES.

ARTICLE VI.—Destroying Nerves. By W. H. Shadoan, D.D.S .... 554

ARTICLE VII.—Cleft Palate .... 556

ARTICLE VIII.—Theory of Nervous Force .... 557

ARTICLE IX.—Arthur's System of Treatment for the Prevention of Decay of the Teeth. By O. A. Jarvis, D.D.S .... 558

ARTICLE X.—Soluble Glass and its Applications .... 565

ARTICLE XI.—Xylol the New Remedy for Small Pox. By C. R. C. Tichborne, F.R.C.S .... 568

### MONTHLY SUMMARY.

Painless Extraction of Teeth .... 569
External Application of Chloral Hydrate .... 570
Facial and Dental Neuralgia .... 570
A Novel Surgical Operation .... 571
Chloral in Toothache .... 571
Cell or Skin Grafting .... 572
A New Styptic .... 572
White Gutta Percha .... 573
Styptic Cotton .... 573

### EDITORIAL DEPARTMENT.

The Cumming's Patent .... 574
Foil Cylinders .... 574
Death from Nitrous Oxide Gas .... 575
Death from Alveolar Hemorrhage .... 576

### OBITUARY.

Dr. B. T. Whitney .... 576

